# Harmonizing Surveillance Methodologies for Group A Streptococcal Diseases^[Author-notes ofac210-FM1]^

**DOI:** 10.1093/ofid/ofac210

**Published:** 2022-09-15

**Authors:** Hannah C Moore, Kate M Miller, Jonathan R Carapetis, Chris A Van Beneden

**Affiliations:** Wesfarmers Centre of Vaccines and Infectious Diseases, Telethon Kids Institute, University of Western Australia, Perth, Western Australia, Australia; Wesfarmers Centre of Vaccines and Infectious Diseases, Telethon Kids Institute, University of Western Australia, Perth, Western Australia, Australia; Wesfarmers Centre of Vaccines and Infectious Diseases, Telethon Kids Institute, University of Western Australia, Perth, Western Australia, Australia; Perth Children’s Hospital, Perth, Western Australia, Australia; Centers for Disease Control and Prevention Foundation, Atlanta, Georgia, USA

**Keywords:** epidemiology, group A *Streptococcus*, *Streptococcus pyogenes*, surveillance

## Abstract

Group A *Streptococcus* (Strep A) is responsible for a significant global health and economic burden. The recent prioritization of Strep A vaccine development by the World Health Organization has prompted global research activities and collaborations. To progress this prioritization, establishment of robust surveillance for Strep A to generate updated regional disease burden estimates and to establish platforms for future impact evaluation is essential. Through the activities of the Strep A Vaccine Global Consortium (SAVAC), we have refined and harmonized surveillance protocols for 7 Strep A disease endpoints with a view that these will form part of surveillance standards for ongoing research and public health activities.

Group A *Streptococcus* (Strep A) is a β-hemolytic, gram-positive bacterium (*Streptococcus pyogenes*) considered to be pathogenic only for humans. Strep A arguably has the broadest clinical spectrum of any infectious pathogen. Diseases include superficial noninvasive infections such as pharyngitis and skin infections (eg, impetigo and cellulitis); invasive diseases such as bacteremia, meningitis, puerperal sepsis, and necrotizing fasciitis; immune- and toxin-mediated diseases such as scarlet fever and streptococcal toxic shock syndrome; and sequelae of immune-mediated diseases such as acute rheumatic fever (ARF), acute poststreptococcal glomerulonephritis, rheumatic heart disease (RHD), and chronic kidney disease [1].

As the disease spectrum associated with Strep A disease is wide and complex, accurately estimating the global burden of disease is challenging. The most recent global burden of Strep A disease study was published in 2005; Strep A was estimated to cause 517 000 deaths each year [2]. In 2019, the inclusion of more recent data for RHD-related deaths revised the 2005 estimate to 639 000 deaths each year and an estimated 618 million new infections annually in addition to 198 million existing cases [3]. In the 2019 update, RHD-related deaths accounted for 467 000 (approximately three-quarters) of all deaths due to Strep A, while deaths due to invasive infection accounted for 163 000 (approximately one-third). A recent review of the burden of RHD has shown a clear negative correlation between sociodemographic index and incidence and prevalence of RHD [4]. Although the mortality of Strep A diseases is borne disproportionately by low- and middle-income countries (LMICs), there remains a significant burden of Strep A diseases (particularly for pharyngitis, impetigo, and invasive infections) in high-income countries (HICs). A contemporary estimate of the full spectrum of Strep A diseases and their sequelae with accurate data from both HICs and LMICs is lacking.

Vaccine development for Strep A is gaining momentum, spurred by the 2018 World Health Assembly that adopted a global resolution on ARF and RHD [5]. Prioritization of vaccine development followed, with the World Health Organization (WHO) publication of the vaccine technical roadmap [6] and preferred product characteristics (PPCs) [7]. Subsequently, the American Heart Association published a position statement in 2020 on RHD, pledging its support for vaccine development [8]. These events have led to the establishment of an international consortium—the Wellcome Trust–funded Strep A Vaccine Global Consortium (SAVAC; https://savac.ivi.int)—to realize the global need for a Strep A vaccine. The mission of SAVAC is to ensure that safe, effective, and affordable Strep A vaccines are available and implemented to decrease the burden of Strep A disease among those most in need. An initial key step in this mission is the acquisition of data needed to design and plan clinical trials to measure vaccine efficacy and safety. Such data requirements include age-specific incidence of key clinical endpoints in well-characterized populations. As guided by the WHO PPCs for Strep A vaccines, these endpoints are pharyngitis and impetigo [7], which are considered the primary intermediates on the causal pathway to immune-mediated and invasive Strep A conditions. Consequently, robust surveillance of these and other Strep A endpoints is crucial in the development of a safe and effective Strep A vaccine.

## SURVEILLANCE STANDARDS

The primary objectives of public health surveillance are to identify and monitor burden of disease over time, to detect trends, to determine disease risk factors and the populations at greatest risk, and to both measure the need for interventions and to monitor the effects of public health interventions. WHO has published surveillance standards providing information for countries to help understand disease burden and inform vaccine policy decisions and recommendations [9]. Although these standards are focused on vaccine-preventable diseases (VPDs), key guiding principles for best-practice surveillance are the same for Strep A and other important public health pathogens for which vaccines are not licensed. Importantly, poor-quality surveillance can be worse than no surveillance at all [9], which can produce misleading data on the burden of disease and the effects—or lack of effect—of interventions. This underscores the need for high-quality surveillance standards, regardless of the sociodemographic setting in which surveillance activities will be implemented. Objectives of such surveillance should be clear. For infectious diseases with vaccines under development or on the near horizon, key objectives include the generation of age-specific trends in disease in the pre-vaccine era that can be compared to the post-vaccine era, providing evidence for new vaccine introduction as well as monitoring changes in diseases strain or types [9]. Additional objectives of disease surveillance may be to monitor the impact of other primordial interventions.

Accurate and accepted case definitions and classifications are a pivotal part of effective surveillance. This can be a challenge for Strep A; some clinical endpoints (eg, scarlet fever, ARF, RHD) are specific to Strep A, while others (eg, cellulitis, pharyngitis) are non-specific and have multiple etiologies. This underscores the importance of clear case definitions, detailed case classifications, and well-defined case ascertainment methodologies when establishing surveillance, especially for non-specific clinical syndromes. A recent example of this is in South Africa where incidence of all-cause pneumonia was 30% lower using an existing surveillance system compared to active surveillance from a prospective birth cohort. The differences in estimated disease incidence were attributable to higher levels of nurse training to detect and classify pneumonia cases in the prospective birth cohort study with active case finding compared to the pre-existing passive surveillance study [10]. While passive surveillance is less costly than active surveillance and more likely to be implemented in settings with limited resources, accurate case definitions and staff training are essential to maximize the benefits and accuracy of this surveillance approach. A recently completed systematic review on global pharyngitis found wide-ranging estimates of incidence largely due to diverse approaches to surveillance; no 2 studies utilized comparable methodologies for case ascertainment and surveillance type [11].

A primary purpose for standardizing surveillance protocols is to allow the resulting data to be compared across multiple studies, diverse geographical locations, and time. This will not only facilitate estimations of the global burden of disease but allow for results of future vaccine efficacy trials to be applied to other jurisdictions with a similar burden of disease. Therefore, one of our motivations for compiling the suite of Strep A surveillance protocols presented in this supplement is to establish surveillance standards that can be incorporated or added to those developed for other diseases and VPDs [9]. The WHO has published numerous standards for VPDs of global importance; we envisage that the Strep A surveillance protocols presented here as stand-alone protocols for each Strep A endpoint can, in the future, be adopted into standards for Strep A surveillance. Protocols that are publicly available and endorsed through WHO and other global organizations can be updated when further advances in microbiological testing methods or data capture systems occur. The overall goal of these surveillance standards is to allow establishment of sustainable and comprehensive surveillance systems targeted for VPDs that consider differences in surveillance strategy by country income level status and capacity for surveillance infrastructure [12]. Development of standardized surveillance also contributes to Strategic Priority 1 of the Immunization Agenda 2030 (IA2030) [13].

## DEVELOPMENT OF STREP A STANDARDIZED PROTOCOLS

In 2008, an international working group of experts in surveillance and Strep A diseases was established with support from the US National Institutes of Health in collaboration with WHO to develop standardized epidemiological surveillance protocols for both acute Strep A diseases (pharyngitis, impetigo, invasive Strep A infections) and their immune sequelae (ARF, RHD, and acute poststreptococcal glomerulonephritis). These surveillance protocols were not widely disseminated and since have become outdated, largely due to advances in diagnostic testing methods. Additionally, not all clinical endpoints of Strep A (such as cellulitis and scarlet fever) were included in these original protocols.

Through the activities of SAVAC, an expert Burden of Disease Working Group (BoDWG) was established comprised of 13 members from 7 geographically diverse countries (representing 5 WHO regions), with expertise covering Strep A and other VPDs, disease surveillance, and vaccine program implementation. Under the guidance of the BoDWG, we updated and expanded the original 2008 surveillance protocols. We established a formal process of review whereby the lead authors of each protocol updated case definitions and case classifications, and incorporated new diagnostic methods in conjunction with the BoDWG. We then engaged with subject-matter experts for each of the 7 key clinical Strep A endpoints: pharyngitis (which incorporates scarlet fever), impetigo, cellulitis, invasive Strep A disease, ARF, RHD, and acute poststreptococcal glomerulonephritis. An iterative review process was undertaken to harmonize each protocol.

## PROTOCOL STRUCTURE

Each protocol follows a consistent structure including surveillance objectives pertinent to each clinical endpoint; case definitions and classifications including microbiological tests for detection of Strep A; methods of case ascertainment and different surveillance settings; and ideal surveillance populations including eligibility criteria. Where appropriate, the protocols also include the following additional surveillance considerations: recommendations for periods of surveillance; seasonality considerations; resources (eg, visual aids and photographs for impetigo) and training needed; discussion of community engagement; suggested treatments (eg, use of antibiotics) to monitor in some surveillance systems; measurement of disease burden (eg, incidence and prevalence); core elements of case reporting forms; and quality assurance and control methods and ethical considerations.

While some sections, such as quality assurance and ethical considerations, are consistent across each clinical endpoint (and are located in the Supplementary Materials of each manuscript), there are important differences in others. While the definitions of active and passive surveillance are consistent, the characteristics of each surveillance system will vary with each Strep A endpoint. For example, a best-practice passive surveillance system for severe disease outcomes for which infected individuals typically seek medical treatment, such as invasive infections, may involve regular review of hospital discharge records. For a milder and often self-limiting disease such as pharyngitis, which has a higher community-level burden of disease, passive surveillance in the primary healthcare setting or a school setting may be appropriate.

## UTILITY OF PROTOCOLS

To facilitate a contemporary global review of the burden of Strep A diseases under the auspices of SAVAC, we developed an innovative systematic framework prioritizing burden of disease data requirements for vaccines with a particular focus on Strep A [14]. We used this framework to identify research priorities to aid in Strep A vaccine development and implementation. Not surprisingly, establishing sentinel surveillance sites for pharyngitis and impetigo was a top research priority. Development of this suite of standardized surveillance protocols will provide important, practical guidance that can be used by public health personnel in diverse country settings to establish or improve surveillance for Strep A and improve disease burden estimates.

As quoted by Foege and colleagues in 1976 [15], “the reason for collecting, analyzing, and disseminating information on a disease is to control that disease. Collection and analysis should not be allowed to consume resources if action does not follow.” A key motivation for updating and harmonizing the 7 surveillance protocols is for their widespread use across numerous geographically and demographically diverse settings. The adoption of these protocols to public health surveillance or research activities will provide consistency in measuring disease burden for all major endpoints of Strep A.

## CONCLUSIONS

In summary, this supplement presents best-practice guidelines to conduct surveillance of multiple clinical endpoints of Strep A, a pathogen responsible for a high global mortality and disease burden. With accelerated efforts of vaccine development for Strep A, we envisage these protocols to have widespread use in helping to accurately capture age-specific incidence and prevalence of Strep A infections, and establish the necessary infrastructure for sites to progress to Strep A vaccine clinical trials when a suitable vaccine candidate becomes available.

## Supplementary Material

ofac210_Supplementary_Data
