# Supplementary material for: Harmonizing Surveillance Methodologies for Group A Streptococcal Diseases
Source: Open Forum Infect Dis. 2022 Sep 15;9(Suppl 1):S1–4. doi: 10.1093/ofid/ofac210 (PMC9474938; doi:10.1093/ofid/ofac210)
Supplement: ofac210_Supplementary_Data [file ofac210_supplementary_data.docx]

**Harmonizing surveillance methodologies for group A streptococcal diseases**

Hannah C Moore^1^, Kate M Miller^1^, Jonathan Carapetis^1,2^, Chris Van Beneden^3^

1 Wesfarmers Centre of Vaccines and Infectious Diseases, Telethon Kids Institute, University of

Western Australia, Perth, Western Australia, Australia

^2^ Perth Children’s Hospital, Perth, Western Australia, Australia

^3^ CDC Foundation, Atlanta, Georgia, USA

*Corresponding Author:*

Hannah C Moore

Wesfarmers Centre for Vaccines and Infectious Diseases, Telethon Kids Institute, University of Western Australia, PO Box 855, West Perth WA, Australia 6872

Email: [hannah.moore@telethonkids.org.au](mailto:hannah.moore@telethonkids.org.au); Phone: +61 409 100 007

*Word count:*

105 (abstract); 1924 (main text)

**Summary**

Group A *Streptococcus* (Strep A) is responsible for a significant global health and economic burden. The recent prioritisation of Strep A vaccine development by the World Health Organization has prompted global research activities and collaborations. To progress this prioritisation, establishment of robust surveillance for Strep A to generate updated regional disease burden estimates and to establish platforms for future impact evaluation is essential. Through the activities of SAVAC, a Strep A global vaccine consortium, we have refined and harmonised surveillance protocols for seven Strep A disease endpoints with a view that these will form part of surveillance standards for ongoing research and public health activities.

**Key words:** surveillance; group A *Streptococcus*; *Streptococcus pyogenes*; epidemiology

Group A *Streptococcus* (Strep A) is a β-haemolytic, Gram-positive bacterium (*Streptococcus pyogenes)* considered to be pathogenic only for humans. Strep A arguably has the broadest clinical spectrum of any infectious pathogen. Diseases include superficial non-invasive infections such as pharyngitis and skin infections (e.g., impetigo and cellulitis); invasive diseases such as bacteraemia, meningitis, puerperal sepsis and necrotising fasciitis; immune- and toxin-mediated diseases such as scarlet fever and streptococcal toxic shock syndrome; and sequelae of immune-mediated diseases such as acute rheumatic fever (ARF), acute post-streptococcal glomerulonephritis, rheumatic heart disease (RHD) and chronic kidney disease [1].

As the disease spectrum associated with Strep A disease is wide and complex, accurately estimating the global burden of disease is challenging. The most recent global burden of Strep A disease study was published in 2005. Strep A was estimated to cause 517,000 deaths each year [2]. In 2019, the inclusion of more recent data for RHD-related deaths revised the 2005 estimate to 639,000 deaths each year and an estimated 618 million new infections annually in addition to 198 million existing cases [3]. In the 2019 update, RHD-related deaths accounted for 467,000 (approximately three-quarters) of all deaths due to Strep A, while deaths due to invasive infection accounted for 163,000 (approximately a third). A recent review of the burden of RHD has shown a clear negative correlation between socio-demographic index and incidence and prevalence of RHD [4]. Although the mortality of Strep A diseases is borne disproportionately by low-middle income countries (LMIC), there remains a significant burden of Strep A diseases (particularly for pharyngitis, impetigo and invasive infections) in high-income countries (HIC). A contemporary estimate of the full spectrum of Strep A diseases and their sequelae with accurate data from both HIC and LMIC is lacking.

Vaccine development for Strep A is gaining momentum, spurred by the 2018 World Health Assembly that adopted a global resolution on ARF and RHD [5]. Prioritisation of vaccine development followed, with the World Health Organization (WHO) publication of the vaccine technical roadmap [6] and preferred product characteristics (PPC) [7]. Subsequently the American Heart Association published a position statement in 2020 on RHD, pledging their support for vaccine development [8]. These events have led to the establishment of an international consortium—the Wellcome Trust funded Strep A Vaccine Consortium, SAVAC ([www.savac.ivi.int/](http://www.savac.ivi.int/)) —to realise the global need for a Strep A vaccine. The mission of SAVAC is to ensure that safe, effective, and affordable Strep A vaccines are available and implemented to decrease the burden of Strep A disease among those most in need. An initial key step in this mission is the acquisition of data needed to design and plan clinical trials to measure vaccine efficacy and safety. Such data requirements include age-specific incidence of key clinical endpoints in well-characterised populations. As guided by the WHO PPC for Strep A vaccines, these endpoints are pharyngitis and impetigo [7], which are considered the primary intermediates on the causal pathway to immune-mediated and invasive Strep A conditions. Consequently, robust surveillance of these and other Strep A endpoints is crucial in the development of a safe and effective strep A vaccine.

**Surveillance standards**

The primary objectives of public health surveillance are to identify and monitor burden of disease over time, detect trends, determine disease risk factors and the populations at greatest risk, and to both measure the need for interventions and to monitor the effects of public health interventions. WHO has published surveillance standards providing information for countries to help understand disease burden and inform vaccine policy decisions and recommendations [9]. Although these standards are focused on vaccine preventable diseases (VPDs), key guiding principles for best-practice surveillance are the same for Strep A and other important public health pathogens for which vaccines are not licensed. Importantly, poor quality surveillance can be worse than no surveillance at all [9], which can produce misleading data on the burden of disease and the effects—or lack of effect—of interventions. This underscores the need for high quality surveillance standards, regardless of the socio-demographic setting that surveillance activities will be implemented in. Objectives of such surveillance should be clear. For infectious diseases with vaccines under development or on the near horizon, key objectives include the generation of age-specific trends in disease in the pre-vaccine era that can be compared to post-vaccine era; providing evidence for new vaccine introduction as well as monitoring changes in diseases strain or types [9]. Additional objectives of disease surveillance may be to monitor the impact of other primordial interventions.

Accurate and accepted case definitions and classifications are a pivotal part of effective surveillance. This can be a challenge for Strep A; some clinical endpoints (e.g., scarlet fever, ARF, RHD) are specific to Strep A, while others (e.g., cellulitis, pharyngitis) are non-specific and have multiple aetiologies. This underscores the importance of clear case definitions, detailed case classifications and well-defined case ascertainment methodologies when establishing surveillance, especially for non-specific clinical syndromes. A recent example of this is in South Africa where incidence of all-cause pneumonia was 30% lower using an existing surveillance system compared to active surveillance from a prospective birth cohort. The differences in estimated disease incidence were attributable to higher levels of nurse training to detect and classify pneumonia cases in the prospective birth cohort study with active case finding compared to the pre-existing passive surveillance study [10]. While passive surveillance is less costly than active surveillance and more likely to be implemented in settings with limited resources, accurate case definitions and staff training are essential to maximise the benefits and accuracy of this surveillance approach. A recently completed systematic review on global pharyngitis found wide ranging estimates of incidence largely due to diverse approaches to surveillance—no two studies utilized comparable methodologies for case ascertainment and surveillance type (Miller et al, publication pending).

A primary purpose for standardising surveillance protocols is to allow the resulting data to be compared across multiple studies, diverse geographical locations, and time. This will not only facilitate estimations of the global burden of disease but allow for results of future vaccine efficacy trials to be applied to other jurisdictions with a similar burden of disease. Therefore, one of our motivations for compiling the suite of Strep A surveillance protocols presented in this Supplement is to establish surveillance standards that can be incorporated or added to those developed for other diseases and VPDs [9]. The WHO has published numerous standards for VPDs of global importance; we envisage that the Strep A surveillance protocols presented here as standalone protocols for each Strep A endpoint can in the future be adopted into standards for Strep A surveillance.. Protocols that are publicly available and endorsed through WHO and other global organisations can be updated when further advances in microbiological testing methods or data capture systems occur. The overall goal of these surveillance standards is to allow establishment of sustainable and comprehensive surveillance systems targeted for VPDs that consider differences in surveillance strategy by country income level status and capacity for surveillance infrastructure [11]. Development of standardised surveillance also contributes to Strategic Priority 1 of the Immunisation Agenda 2030 (IA2030) [12].

**Development of Strep A standardised protocols**

In 2008, an international working group of experts in surveillance and Strep A diseases was established with support from the US National Institutes of Health in collaboration with WHO to develop standardised epidemiological surveillance protocols for both acute Strep A diseases (pharyngitis, impetigo, invasive Strep A infections) and their immune sequelae (ARF, RHD and acute post-streptococcal glomerulonephritis). These surveillance protocols were not widely disseminated and since have become outdated, largely due to advances in diagnostic testing methods. Additionally, not all clinical endpoints of Strep A (such as cellulitis and scarlet fever) were included in these original protocols.

Through the activities of SAVAC, an expert Burden of Disease Working Group (BoDWG) was established comprising of 13 members from seven geographically diverse countries (representing five WHO regions), with expertise covering Strep A and other VPDs, disease surveillance and vaccine program implementation. Under the guidance of the BoDWG, we updated and expanded the original 2008 surveillance protocols. We established a formal process of review whereby the lead authors of each protocol updated case definitions and case classifications, and incorporated new diagnostic methods in conjunction with the BoDWG. We then engaged with subject matter experts for each of the seven key clinical Strep A endpoints: pharyngitis (which incorporates scarlet fever), impetigo, cellulitis, invasive Strep A disease, ARF, RHD and acute post-streptococcal glomerulonephritis. An iterative review process was undertaken to harmonise each protocol.

**Protocol Structure**

Each protocol follows a consistent structure including surveillance objectives pertinent to each clinical endpoint; case definitions and classifications including microbiological tests for detection of Strep A; methods of case ascertainment and different surveillance settings; and ideal surveillance populations including eligibility criteria. Where appropriate, the protocols also include the following additional surveillance considerations: recommendations for periods of surveillance, seasonality considerations, resources (e.g., visual aids and photographs for impetigo) and training needed, discussion of community engagement; suggested treatments (e.g., use of antibiotics) to monitor in some surveillance systems; measurement of disease burden (e.g., incidence and prevalence); core elements of case reporting forms; and quality assurance and control methods and ethical considerations.

While some sections, such as quality assurance and ethical considerations, are consistent across each clinical endpoint (and are located in Supplementary Material), there are important differences in others. While the definitions of active and passive surveillance are consistent, the characteristics of each surveillance system will vary with each Strep A endpoint. For example, a best-practice passive surveillance system for severe disease outcomes for which infected individuals typically seek medical treatment, such as invasive infections, may involve regular review of hospital discharge records. For a milder and often self-limiting disease like pharyngitis which has a higher community-level burden of disease, passive surveillance in the primary healthcare setting or a school setting may be appropriate.

**Utility of protocols**

To facilitate a contemporary global review of the burden of Strep A diseases under the auspices of SAVAC, we developed an innovative systematic framework prioritising burden of disease data requirements for vaccines with a particular focus on Strep A (Moore et al, pending publication). We used this framework to identify research priorities to aid in Strep A vaccine development and implementation. Not surprisingly, establishing sentinel surveillance sites for pharyngitis and impetigo was a top research priority. Development of this suite of standardised surveillance protocols will provide important, practical guidance that can be used by public health personnel in diverse country settings to establish or improve surveillance for Strep A and improve disease burden estimates.

As quoted by Foege and colleagues in 1976 [13], “*the reason for collecting, analyzing, and disseminating information on a disease is to control that disease. Collection and analysis should not be allowed to consume resources if action does not follow.*” A key motivation for updating and harmonising the seven surveillance protocols is for their widespread use across numerous geographically and demographically diverse settings. The adoption of these protocols to public health surveillance or research activities will provide consistency in measuring disease burden for all major endpoints of Strep A.

**Conclusion**

In summary, this Supplement presents best practice guidelines to conduct surveillance of multiple clinical endpoints of Strep A—a pathogen responsible for a high global mortality and disease burden. With accelerated efforts of vaccine development for Strep A, we envisage these protocols to have widespread use in helping to accurately capture age-specific incidence and prevalence of Strep A infections, and establish the necessary infrastructure for sites to progress to Strep A vaccine clinical trials when a suitable vaccine candidate becomes available.

**ACKNOWLEDGMENTS**

**Financial support**

The supplement presents and includes work from the Strep A Vaccine Global Consortium, SAVAC ([https://savac.ivi.int/](https://eur01.safelinks.protection.outlook.com/?url=https%3A%2F%2Fsavac.ivi.int%2F&data=04%7C01%7CD.King%40wellcome.org%7C2d581d5efe0e48d5843508d99a282220%7C3b7a675a1fc84983a100cc52b7647737%7C0%7C0%7C637710319424023200%7CUnknown%7CTWFpbGZsb3d8eyJWIjoiMC4wLjAwMDAiLCJQIjoiV2luMzIiLCJBTiI6Ik1haWwiLCJXVCI6Mn0%3D%7C1000&sdata=OPjyW8zPaWJGxOe1gO7ZNA%2F6pM8SUURkAiOiUJaJPrc%3D&reserved=0)). SAVAC is administered through International Vaccine Institute and has received funding from the Wellcome Trust (215490/Z/19/Z).

**Potential conflicts of interest**

HCM has received funding from Merck Sharp and Dohme under the Merck Investigator Studies Program (not related to the work presented in this article). All other authors declare no competing interests.

**REFERENCES**

1. Cannon JW, Jack S, Wu Y, et al. An economic case for a vaccine to prevent group A streptococcus skin infections. *Vaccine* 2018;36(46):6968-78. doi: 10.1016/j.vaccine.2018.10.001 [published Online First: 2018/10/21]

2. Carapetis JR, Steer AC, Mulholland EK, et al. The global burden of group A streptococcal diseases. *Lancet Infect Dis* 2005;5(11):685-94. doi: 10.1016/s1473-3099(05)70267-x

3. Hand RM, Snelling TL, Carapetis JR. 40 - Group A Streptococcus. In: Ryan ET, Hill DR, Solomon T, et al., eds. Hunter's Tropical Medicine and Emerging Infectious Diseases (Tenth Edition). London: Elsevier 2020:429-38.

4. Lv M, Jiang S, Liao D, et al. Global Burden of Rheumatic Heart Disease and its Association with Socioeconomic Development Status, 1990–2019. *Eur J Prev Cardiol* 2022

5. White A. WHO Resolution on rheumatic heart disease: Oxford University Press, 2018.

6. World Health Organization. Group A S*treptococcus* vaccine development technology roadmap: priority activities for development, testing, licensure and global availability of group A S*treptococcus* vaccines. Geneva: World Health Organization, 2018.

7. World Health Organization. WHO Preferred Product Characteristics for Group A Streptococcus Vaccines. Geneva: World Health Organization, 2018.

8. Beaton A, Kamalembo FB, Dale J, et al. The American Heart Association’s Call to Action for Reducing the Global Burden of Rheumatic Heart Disease: A Policy Statement From the American Heart Association. *Circulation* 2020;142(20):e358-e68.

9. World Health Organization. Overview of VPD Surveillance Principles 2018 [1-46]. Available from: <https://cdn.who.int/media/docs/default-source/immunization/vpd_surveillance/vpd-surveillance-standards-publication/who-surveillancevaccinepreventable-01-overview-r2.pdf?sfvrsn=932d9e08_8&download=true> accessed 10 Jan 2022.

10. Le Roux DM, Myer L, Nicol MP, et al. Incidence of childhood pneumonia: facility-based surveillance estimate compared to measured incidence in a South African birth cohort study. *BMJ open* 2015;5(12):e009111.

11. World Health Organization. Global strategy for comprehensive Vaccine-Preventable Disease (VPD) surveillance 2020 [1-20]. Available from: <https://www.who.int/publications/m/item/global-strategy-for-comprehensive-vaccine-preventable-disease-(vpd)-surveillance> accessed 10 Jan 2022.

12. World Health Organization. Immunization Agenda 2030: A global strategy to leave no one behind 2020 [Available from: <https://www.who.int/teams/immunization-vaccines-and-biologicals/strategies/ia2030> accessed 1 April 2020.

13. Foege WH, Hogan RC, NEWTON LH. Surveillance projects for selected diseases. *Int J Epidemiol* 1976;5(1):29-37.
